# Identification of Potential Biomarkers for Thyroid Cancer Using Bioinformatics Strategy: A Study Based on GEO Datasets

**DOI:** 10.1155/2020/9710421

**Published:** 2020-04-01

**Authors:** Yujie Shen, Shikun Dong, Jinhui Liu, Liqing Zhang, Jiacheng Zhang, Han Zhou, Weida Dong

**Affiliations:** ^1^Department of Otorhinolaryngology, The First Affiliated Hospital of Nanjing Medical University, Nanjing, 210029 Jiangsu, China; ^2^Department of Gynecology, The First Affiliated Hospital of Nanjing Medical University, Nanjing, 210029 Jiangsu, China

## Abstract

**Background:**

The molecular mechanisms and genetic markers of thyroid cancer are unclear. In this study, we used bioinformatics to screen for key genes and pathways associated with thyroid cancer development and to reveal its potential molecular mechanisms.

**Methods:**

The GSE3467, GSE3678, GSE33630, and GSE53157 expression profiles downloaded from the Gene Expression Omnibus database (GEO) contained a total of 164 tissue samples (64 normal thyroid tissue samples and 100 thyroid cancer samples). The four datasets were integrated and analyzed by the RobustRankAggreg (RRA) method to obtain differentially expressed genes (DEGs). Using these DEGs, we performed gene ontology (GO) functional annotation, pathway analysis, protein-protein interaction (PPI) analysis and survival analysis. Then, CMap was used to identify the candidate small molecules that might reverse thyroid cancer gene expression.

**Results:**

By integrating the four datasets, 330 DEGs, including 154 upregulated and 176 downregulated genes, were identified. GO analysis showed that the upregulated genes were mainly involved in extracellular region, extracellular exosome, and heparin binding. The downregulated genes were mainly concentrated in thyroid hormone generation and proteinaceous extracellular matrix. Pathway analysis showed that the upregulated DEGs were mainly attached to ECM-receptor interaction, p53 signaling pathway, and TGF-beta signaling pathway. Downregulation of DEGs was mainly involved in tyrosine metabolism, mineral absorption, and thyroxine biosynthesis. Among the top 30 hub genes obtained in PPI network, the expression levels of FN1, NMU, CHRDL1, GNAI1, ITGA2, GNA14 and AVPR1A were associated with the prognosis of thyroid cancer. Finally, four small molecules that could reverse the gene expression induced by thyroid cancer, namely ikarugamycin, adrenosterone, hexamethonium bromide and clofazimine, were obtained in the CMap database.

**Conclusion:**

The identification of the key genes and pathways enhances the understanding of the molecular mechanisms for thyroid cancer. In addition, these key genes may be potential therapeutic targets and biomarkers for the treatment of thyroid cancer.

## 1. Introduction

Thyroid cancer, a most common endocrine cancer, accounts for 1-2% of human tumors. In recent years, the incidence of thyroid cancer, ranking the seventh among all tumors and the fifth for women, has been increasing. The appropriate treatment plan (surgery, radiotherapy, chemotherapy) is designed according to the stage of the disease. Most patients have a good prognosis after percutaneous transluminal coronary angioplasty, but invasive tumor or distant metastases would occur in a small number of patients following this treatment [1]. Therefore, finding key biomarkers and therapeutic targets of thyroid cancer will greatly improve the quality of life for patients.

Since the invention of gene expression microarray technology about 20 years ago, many mRNA profiling data sets have been generated for different biological processes in organisms. Currently, more than 30,000 series and 1 million array-based gene expression data samples are stored in GEO database of the National Center for Biotechnology Information (NCBI) [2]. Microarray analysis is a new method to study tumor genes, search for molecular targets of tumor drug therapy and monitor prognosis. However, due to the heterogeneity of the experimental samples, different detection platforms and data processing methods generate inconsistent results [3]. The study was based on biological information in the GEO database, and the final results were obtained by analyzing the molecular functions of mRNA associated with thyroid cancer and the signal pathways involved.

To more accurately identify DEGs associated with TCHA, bioinformatics methods were applied to analyze datasets from GEO and then the results in this study were integrated. Bioinformatic analyses were performed on these DEGs. Here, we aimed to explore a reliable basis for exploring the molecular mechanisms of THCA pathogenesis and the identification of molecular targets for clinical diagnosis or treatment.

## 2. Materials and Methods

### 2.1. Microarray Data

The GEO database (https://www.ncbi.nlm.nih.gov/geo/) was searched using the keywords “thyroid cancer”. The GSE3467 (including 9 normal thyroid tissue samples and 9 thyroid cancer samples, [4]), GSE3678 (including 7 normal thyroid tissue samples and 7 thyroid cancer samples0, GSE33630 (including 45 normal thyroid tissue samples and 60 thyroid cancer samples, [5]) and GSE53157 (including 3 normal thyroid tissue samples and 24 thyroid cancer samples, [6]) gene expression profile matrix files were downloaded. The data set information is shown in Table 1 and the clinical information is provided in the supplementary materials ([Supplementary-material supplementary-material-1]). We downloaded the four series of matrix TXT files and the corresponding platform TXT files. The gene probe ID in each matrix file was converted to the gene symbol in the platform file by the Perl language. Impute and Limma packages in R software (version: x64 3.2.1) were applied to perform the background correction, normalization and log2 conversion for the matrix data of each GEO dataset.

### 2.2. Data Processing and Identification of DEGs

Differential expression analysis of the each dataset was carried out using the limma package. We defined |log(FC)| ≥ 1 and adj. *P*-val< 0.05 as DEGs screening criteria for thyroid cancer samples from four microarray datasets, and generated heat maps and volcano maps of each dataset. In addition, a txt file of all gene lists sorted by log (FC) in each data set was saved for the subsequent integration analysis.

### 2.3. Integration of Microarray Data

We downloaded the RobustRankAggreg (RRA) package and used the R software to run the instruction code. The four lists of genes ranked by expression level were integrated using the RRA package in R software. The RRA method was based on the assumption that all genes were unordered in each list. Equally, |log(FC)| ≥ 1 and adj. *P*-val< 0.05 were considered statistically significant for the DEGs. As a result, a post-integration heat map was generated and the upregulated and downregulated genes were further screened for further analysis. All the R packages performed in our research were arranged in R software.

### 2.4. Functional Enrichment Analysis of DEGs

The Database for Annotion, Visualition and Intrgrated Discovery (DAVID) 6.8 (https://david.ncifcrf.gov), is an online bioinformatics database for gene functional analysis that integrates biological data and analysis tools, and annotates biological functions for large-scale gene or protein lists. We used DAVID to perform GO analysis on the up-regulated and down-regulated DEGs. The difference was considered statistically significant when adj. *P*-val< 0.05. Subsequently, we introduced the up-regulated and down-regulated differential genes into Cytoscape's plug-in ClueGO for pathway analysis. Similarly, the difference was considered statistically significant when adj. *P*-val ≤ 0.05.

### 2.5. PPI Network Construction

STRING (http://string-db.org) was used to analyze the direct (physical) and indirect (function) associations between different genes. Protein-Protein Interaction (PPI) network analysis was conducted on all integrated DEGs by STRING10.0 with the effective binding score set >0.7 [7]. Then the results were imported into Cytoscape 3.7.1 to build a network model. Using Cytoscape's plugin Cytohubba, we selected the top 30 DEGs with high connectivity in the gene expression network as the hub genes according to the degree algorithm.

### 2.6. Survival Analysis of Hub Genes

To further clarify the relationship between the hub gene expression and thyroid cancer prognosis, we used Gene Expression Profiling Interactive Analysis (GEPIA, http://gepia.cancer-pku.cn) for survival and statistical analyses using log rank [8]. The difference was considered statistically significant when *P* < 0.05. Due to their importance to the prognosis of thyroid cancer, these hub genes were taken as key genes.

### 2.7. Verification of Key Genes in TCGA

Gene Expression Profiling Interactive Analysis(GEPIA), is a web-based tool (http://gepia.cancer-pku.cn) that delivers fast and customizable functionalities based on TCGA and GTEx data. In this study, we used GEPIA to verify the key genes and the expression of key genes in TCGA was shown by boxplot. The following screening criteria were set as follows: |log(FC)| ≥ 1 and *P*-val< 0.05.

### 2.8. Immunohistochemical Analysis

Due to the high specificity of the binding between antibodies and antigens, we combined the antibody-based approach with transcriptomics data to summarize the global expression profile [9]. The protein expressions of key genes in normal tissues and thyroid cancer tissues were observed using the human protein map (HPA, https://www.proteinatlas.org), a unique collection of antibodies mapped across the entire human proteome by immunohistochemistry and immunocytochemistry.

### 2.9. Identification of Candidate Small Molecules

The Connectivity Map database (CMap, http://www.broadinstitute.org/cmap/), a reference database containing drug-specific gene expression profiles, can be used to discover the connections between small molecules that share a mechanism of action, chemicals and physiological processes, as well as diseases and drugs by submitting the genes that are potentially associated with a particular disease [10, 11]. We matched the DEGs screened from GEO with the data from CMap to predict small molecules that might reverse the biological status of thyroid cancer. The DEGs were divided into the upregulated group and the downregulated group. Then two lists of gene IDs were introduced into CMap for gene set enrichment analysis to obtain small molecules with enrichment values of -1 to +1. The positive connectivity value (close to +1) indicated that the corresponding small molecule could induce gene expression in thyroid cancer, while the negative connectivity value (close to -1) indicated greater similarity between the gene and the small molecule, the potential to reverse the state of thyroid cancer cells. The results were ranked by *P* values. We chose the top 4 small molecules and analyzed their 3D conformations in PubChem (http://www.pubchem.ncbi.nlm.gov), a collection of chemical information, including substance information, compound structures, and biological activities.

## 3. Results

### 3.1. Identification of DEGs

**Figure 1:**
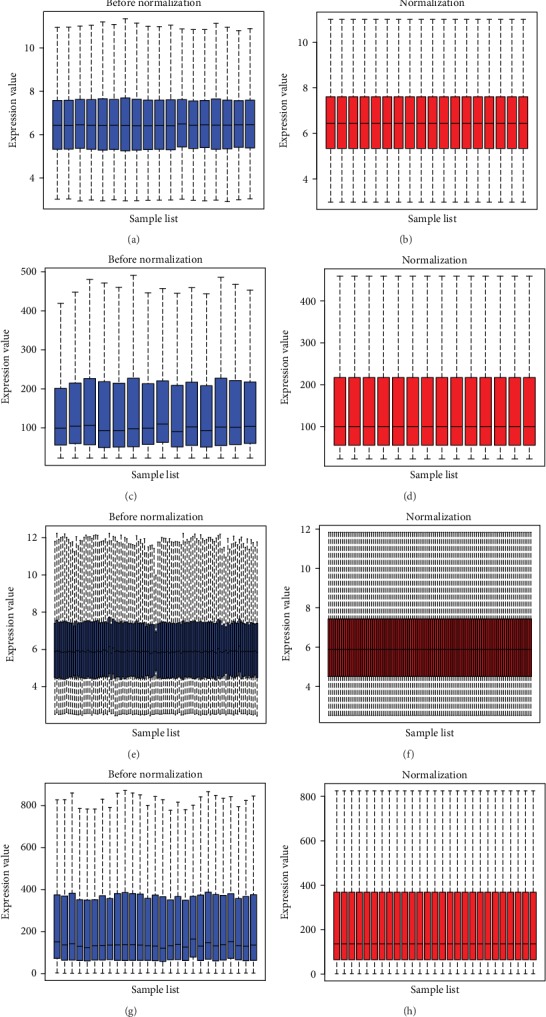
Normalization of datasets. (a–b) Normalization of GSE3467 (c–d) Normalization of GSE3678. (e–f) Normalization of GSE33630. (g–h) Normalization of GSE53157. Blue: data before normalization. Red: data after normalization.

**Figure 2:**
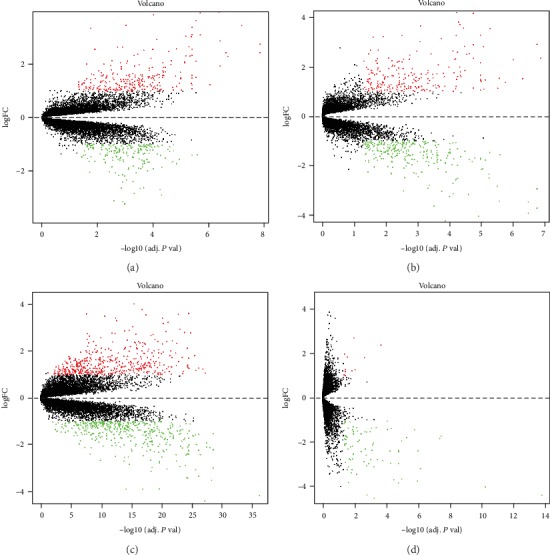
Volcano plots of each dataset. (a) GSE3467, (b) GSE3678, (c) GSE33630, (d) GSE53157. Red dots: upregulated DEGs. Green dots: downregulated DEGs. Black dpots: genes with no significant difference in expression.

**Figure 3:**
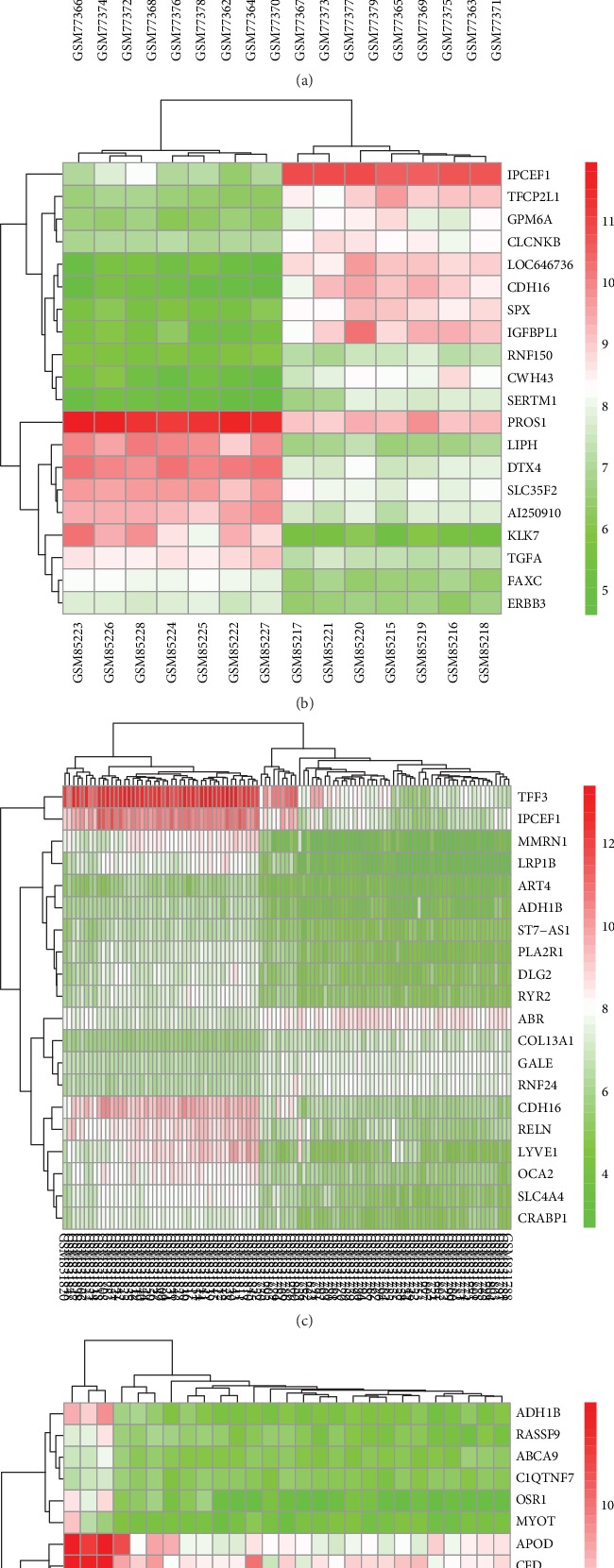
Cluster heat maps of each dataset. (a) GSE3467, (b) GSE3678, (c) GSE33630, (d) GSE53157. Red: relatively upregulated DEGs; Green: relatively downregulated DEGs.

The thyroid cancer expression profile chip data sets GSE3467, GSE3678, GSE33630 and GSE53157 were normalized (Figure 1). The GSE3467 dataset contained 501 differential genes, including 282 upregulated genes and 219 downregulated genes. The GSE3678 dataset contained 526 differential genes, including 226 upregulated genes and 300 downregulated genes. The GSE33630 dataset contained 904 differential genes, including 481 upregulated genes and 423 downregulated genes. The GSE53157 dataset contained 93 differential genes, including 11 upregulated genes and 82 downregulated genes. The volcano plots of each dataset are shown in Figure 2, and the cluster heat maps of the top 20 DEGs in each dataset are shown in Figure 3.

### 3.2. Identification of Integrated DEGs

**Figure 4:**
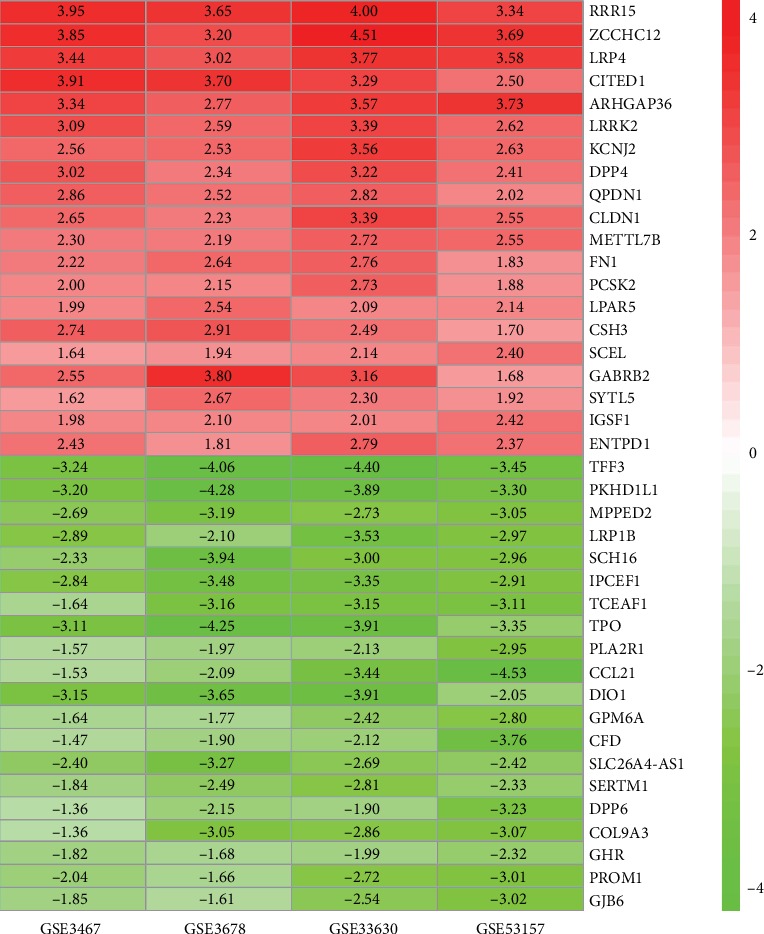
Heatmap of the top 20 upregulated and downregulated DEGs in the integrated analysis. Red: relatively upregulated genes. Green: relatively downregulated genes. Value in the box: log FC value.

A total of 330 integrated DEGs, including 154 upregulated genes and 176 downregulated genes, were identified by the RRA method.  Table 2 shows the DEGs.  Figure 4 presents the heat map of the top 20 upregulated and downregulated integrated DEGs.

### 3.3. GO Enrichment Analysis of DEGs

**Figure 5:**
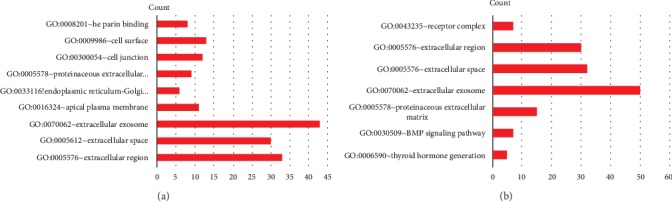
GO terms of integrated DEGs. (a) Significant GO terms of upregulated DEGs. (b) Significant GO terms of downregulated DEGs.

GO function annotations of the integrated DEGs were mainly divided into three parts: biological process (BP), cell composition (CC) and molecular function (MF). As shown in Figure 5 and Table 3, the upregulated DEGs were mainly concentrated in the extracellular region (ontology: CC), extracellular exosome (ontology: CC), and heparin binding (ontology: MF); the downregulated DEGs were mainly concentrated in thyroid hormone generation (ontology: BP) and proteinaceous extracellular matrix (ontology: CC).

### 3.4. Functional Enrichment Analysis of DEGs

**Figure 6:**
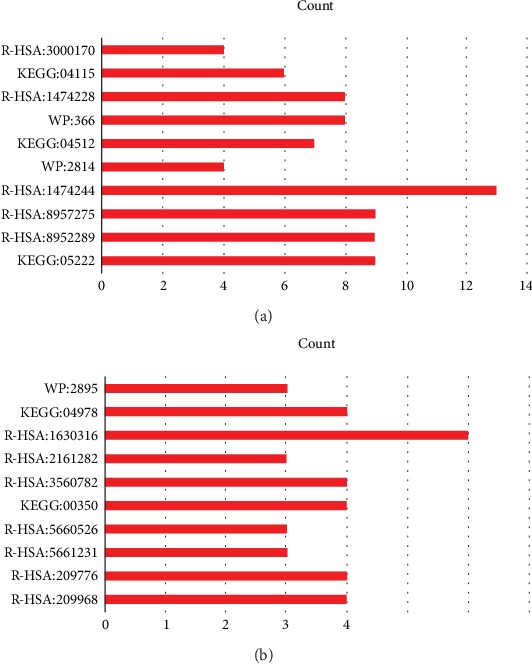
Pathways of integrated DEGs. (a) Significant pathways of upregulated DEGs. (b) Significant pathways of downregulated DEGs.

Functional enrichment analysis of integrated DEGs showed the upregulated DEGs were mainly attached to ECM-receptor interaction, p53 signaling pathway, TGF-beta signaling pathway and other pathways; the downregulated DEGs were mainly attached to tyrosine metabolism, mineral absorption, and thyroxine biosynthesis (Figure 6, Table 4).

### 3.5. PPI Analysis of DEGs

**Figure 7:**
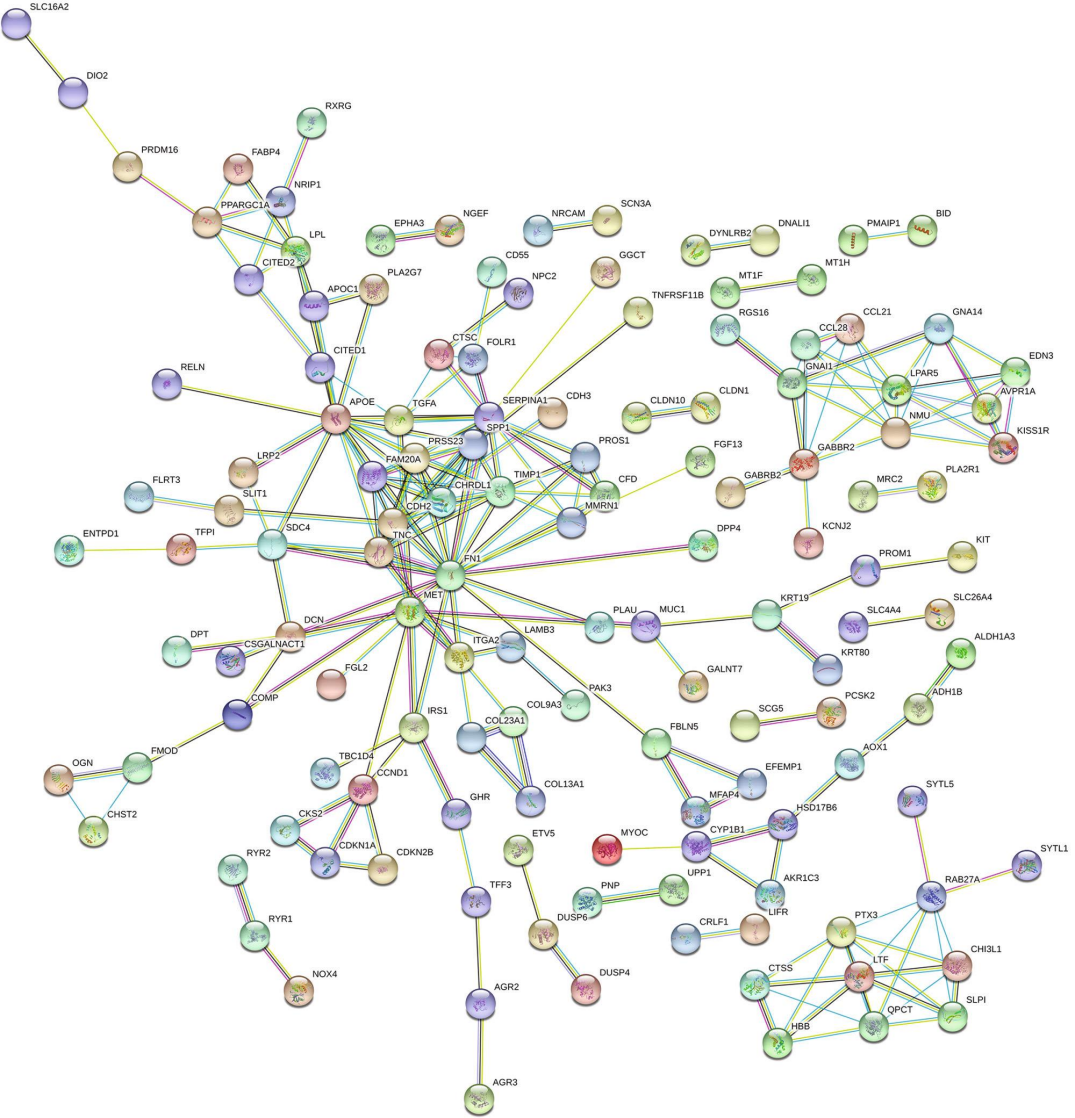
PPI network of integrated DEGs by STRING. Circles: genes. Lines: protein interaction between genes. Results within the circle: structure of proteins. Line color: evidence of the interaction between the proteins.

**Figure 8:**
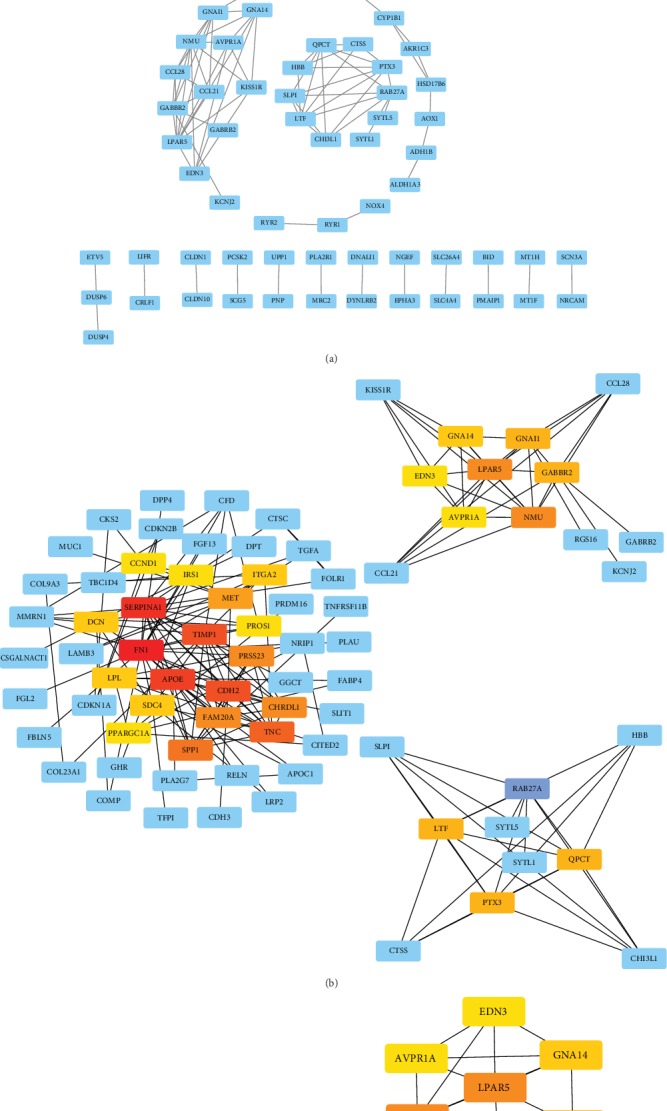
PPI analysis of integrated DEGs based on Sytoscape. (a) Visualization analysis of protein interaction network of all DEGs. (b) Top 30 genes with the highest degree scores. (c) Interconnection of 30 hub genes; darker color representing a higher degree score.

The STRING online database was used to analyze the integrated DEGs obtained after screening and a PPI network was construct (Figure 7). The effective binding fraction was set to be greater than 0.7, which resulted in 313 nodes and 228 edges. The top 30 hub genes were screened out by cytoHubba (Figure 8).

### 3.6. Survival Analysis of Hub Genes

**Figure 9:**
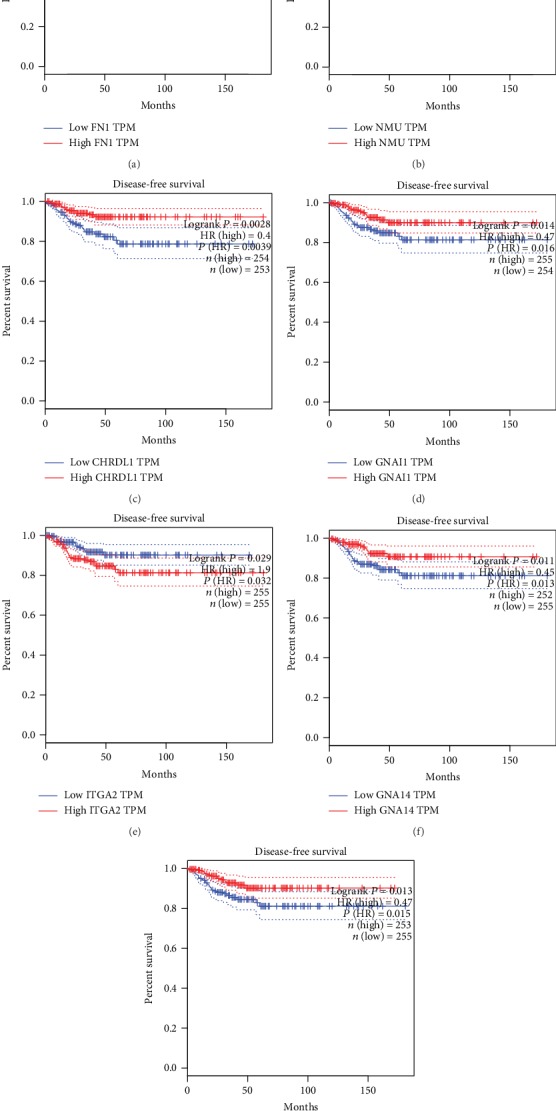
Survival analysis of hub genes in thyroid cancer. (a) FN1. (b) NMU. (c) CHRDL1. (d) GNAI1. (e) ITGA2. (f) GNA14. (g) AVPR1A.

We used GEPIA to analyze the correlation between hub gene expression and thyroid cancer prognosis. It was found that the Disease Free Survival (DFS) was lower in the high FN1 expression group than the low FN1 expression group (*P* = 0.024); the high NMU expression group had lower DFS than the low NMU expression group (*P* = 0.021); the low CHRDL1 expression group had lower DFS than the high CHRDL1 expression group (*P* = 0.0028); the low GNAI1 expression group had lower DFS than the high GNAI1 expression group (*P* = 0.014); the high ITGA2 expression group had lower DFS than the low ITGA2 expression group (*P* = 0.029); the low GNA14 expression group had lower DFS than the high GNA14 expression group (*P* = 0.011). The low AVPR1A expression group had lower DFS than the high AVPR1A expression group (*P* = 0.013). As shown in Figure 9, the other hub genes were not significantly associated with the prognosis of thyroid cancer (*P* > 0.05). Hence, we derived seven key genes related to the prognosis of thyroid cancer: FN1, NMU, CHRDL1, GNAI1, ITGA2, GNA14, AVPR1A.

### 3.7. Verification of Key Genes in TCGA

**Figure 10:**
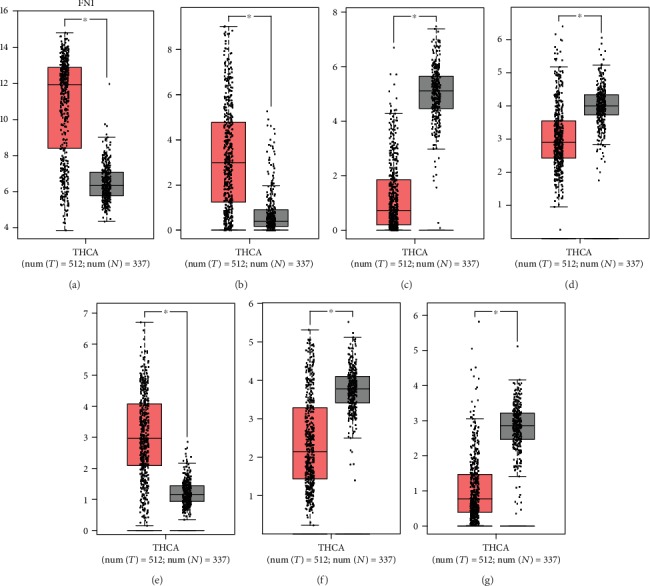
The verification of mRNA expression of key genes in the term of boxplot. (a) FN1. (b) NMU. (c) CHRDL1. (d) GNAI1. (e) ITGA2. (f) GNA14.(g) AVPR1A.

We validated the reliability of the key genes using GEPIA. The databases showed that the key genes were differentially expressed in normal thyroid tissue samples and thyroid cancer samples (Figure 10). By reviewing the original data, we found that the results were consistent with our study.

### 3.8. Immunohistochemical Analysis

**Figure 11:**
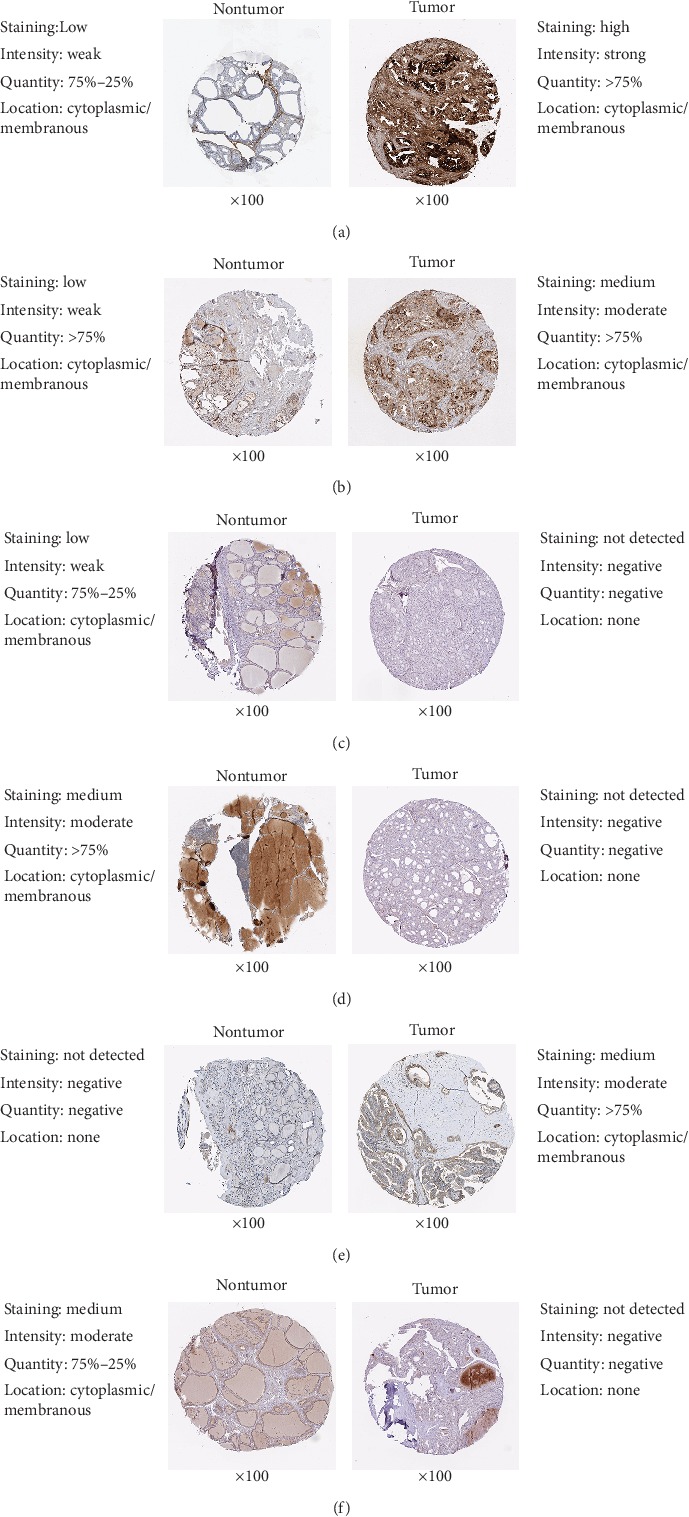
Comparison of protein expression of key genes between normal thyroid tissues and thyroid cancer tissues. (a) FN1. (b) NMU. (c) CHRDL1. (d) GNAI1. (e) ITGA2. (f) GNA14.

The Human Protein Atlas (HPA)-based Immunohistochemistry (IHC) database showed that FN1, NMU, and ITGA2 were upregulated in thyroid cancer tissues compared with normal tissues, while CHRDL1, GNAI1 and GNA14 were downregulated in thyroid cancer tissues (Figure 11). These results confirmed our findings. However, we did not find the association between AVPR1A and thyroid cancer in this database. According to our analysis, we predicted that AVPR1A might also be associated with the development of thyroid cancer, but experimental data were needed to confirm this specific link.

### 3.9. Screening of Small Molecule Drugs Screening

**Figure 12:**
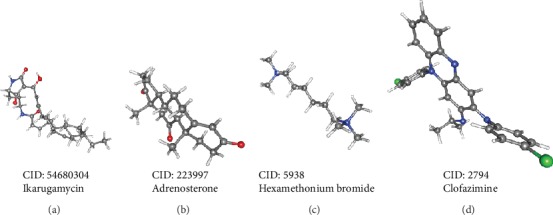
3D conformer of 4 most significant small molecule drugs. (a) Ikarugamycin. (b) Adrenosterone. (c) Hexamethonium bromide. (d) Clofazimine.

In order to find drugs involved in the treatment or prognosis of thyroid cancer, we uploaded the selected DEGs (divided into the upregulated and downregulated groups) to the CMap database and then matched them with small molecule therapy. As shown in Table 5, among the top 4 small molecules, adrenosterone (enrichment value -0.914) and hexamethonium bromide (enrichment value -0.813) exhibited highly significant negative scores. The 3D conformer is shown in Figure 12. These small molecule drugs could reverse the gene expression induced by thyroid cancer. Of course, more experimental data are needed to confirm the potential of these candidate small molecules in treating thyroid cancer.

## 4. Discussion

Although thyroid cancer is a least fatal human cancer, its rising incidence imposes a great burden on both the society and individuals. Since the 1990s, the incidence of thyroid cancer has been growing faster than the other tumors in the United States. The rising morbidity and mortality of thyroid cancer are related to overdiagnosis [12]. Therefore, studying the biomarkers and precise targets associated with the development of thyroid cancer will improve the diagnostic accuracy and thus lessen the economic burden.

In this study, bioinformatics methods were applied to analyze the GSE3467, GSE3678, GSE33630 and GSE53157 datasets from GEO, and GO and pathway analyses were performed in the integrated DEGs. We used STRING to structure the Protein-Protein Interaction (PPI) network of integrated DEGs based on the functional association. Interactions are derived from seven sources: (1) the experiments channel; (2) the database channel; (3) the textmining channel; (4) the coexpression channel; (5) the neighborhood channel; (6) the fusion channel; (7) the co-occurrence channel. Then we applied Cytoscape to identify 30 hub genes. Finally, 7 key genes related to thyroid cancer prognosis were obtained. Among them, FN1, NMU and ITGA2 were upregulated DEGs, while CHRDL1, GNAI1 and GNA14 were downregulated ones. At the same time, gene expression was verified through GEPIA and the level of gene expression protein was verified by HPA.

Fibronectin (FN1), produced by fibroblasts and tumor cells, is a high molecular weight glycoprotein component of the extracellular matrix in the tumor microenvironment (TME). Normally, FN1 supports the cell-extracellular matrix interaction and participates in cell adhesion, migration, metastasis, proliferation and differentiation, as well as embryogenesis, blood clotting, wound healing, development, and tissue homeostasis [13]. Tumor-associated fibroblasts are the most abundant cells in the tumor stroma, and their ability to contract the matrix and induce cancer cell invasion has been well certified. FN1, derived from tumor-associated fibroblasts, stimulates cancer cell invasion after assembly [14]. In addition, as an intrinsic component of epithelial-mesenchymal transition (EMT) regulatory circuit, FN1 is a potential target for interfering EMT during tumorigenesis [15]. Here, we suspect that FN1 is related to the pathway of ECM-receptor interaction. FN1 has been found to be highly expressed in esophageal and cervical cancers and associated with their prognosis [16–18]. In the present study, FN1, as a hub gene with the highest correlation with thyroid cancer, was highly expressed in tumor tissues compared with the adjacent non-tumor or normal tissues. Further validation of the association between FN1 and the development of thyroid cancer may provide new targets for treating thyroid cancer.

As the gene with the second highest correlation with thyroid cancer in our study, neuromedin U (NMU), a neuropeptide originally isolated from the spinal cord of pigs, has multiple physiological functions and is involved in obesity and inflammation [19]. NMU has been confirmed to confer alectinib resistance in non-small cell lung cancer (NSCLC) [20]. Although no experimental data has suggested a direct link between NMU and thyroid cancer, NMU has been shown to promote the development of various tumors, including breast cancer. Garczyk S et al. have demonstrated that NMU may contribute to the progression of NMUR2-positive breast cancer [21] and enhance resistance to tumor immune responses in breast cancers with HER2 overexpression [22], suggesting NMU is a potential drug target for personalized strategies. Furthermore, NMU is involved in the development of endometrial, colorectal and gastric cancers, as well as acute myeloid leukemia caused by TP53 mutations [23–26].

CHRDL1 is a secreted protein and antagonist of bone morphogenetic protein (BMP) that antagonizes BMP-4 activity and induces differentiation of spinal cord-derived neural stem cells into neurons [27]. Pei YF et al. have reported that CHRDL1 expression is significantly downregulated in gastric cancer tissues and associated with low survival. In vitro, CHRDL1 knockdown promotes tumor cell proliferation by activating AKT, ERK and *β*-catenin and boosts tumor cell migration through BMPR II. In vivo experiments have confirmed that CHRDL1 is a tumor suppressor gene that inhibits tumor growth and metastasis [28]. Cyr-Depauw C et al. have found that CHRDL1 is a negative regulator of malignant breast cancer phenotype and can inhibit BMP signal transduction [29]. Since no research has proved that CHRDL1 is related to thyroid cancer, more research is needed to verify the association between CHRDL1 and thyroid cancer and evaluate CHRDL1 as a target for thyroid cancer treatment.

The GO annotations of GNAI1 (G protein subunit *α*i1) include GTP binding and outdated signal transduction activity. The guanine nucleotide binding protein (G protein) acts as a transduction downstream of the G protein coupled receptor (GPCR) in many signaling cascades. The alpha chain, which contains a guanine nucleotide binding site, alternates between active and inactive GTP binding states. Activation of GPCRs promotes GDP release and GTP binding. The alpha subunit has low GTPase activity, which converts the bounding of GTP to GDP, and thereby terminates the signal. Both GDP release and GTP hydrolysis are regulated by regulatory proteins [30, 31]. Cell migration involves a cycle of adhesion and de-adhesion, and the dynamic balance between adhesion and reversal of adhesion regulated by G protein is very important for this process [32]. Cell migration of normal cells is tightly regulated. However, tumor cells are exposed to a modified microenvironment that promotes cell migration [33]. Changes in G protein affect the tumor cell migration.

ITGA2, the integrin subunit *α*2, encodes a protein that forms a heterodimer with the beta subunit and mediates the adhesion of platelets and other cell types to the extracellular matrix. The ITGA2-related pathways include beta-adrenergic signaling and the blood-brain barrier pathway. GO annotations associated with this gene include protein heterodimerization activity and integrin binding. Chernaya G et al. observed higher levels of ITGA2 gene expression in papillary thyroid carcinoma (PTC) tissues compared to normal thyroid tissues [34]. Yang Z et al. found that ITGA2 was a direct target of miR-16, and the down-regulation of miR-16 in invasive PTC resulted in up-regulated ITGA2 gene expression. The high expression level of ITGA2 might lead PTC invasion and migration [35].

GNA14 (G protein subunit *α*14) encodes a member of the guanine nucleotide binding or G protein family. GNA14 mutations induce morphological changes in cells and make cell growth factors independent by upregulating the MAPK pathway. A study found that GNA14 mutations could cause vascular tumors in children [36].

Arginine vasopressin receptor 1A (AVPR1A) is involved in mediation of cell contraction and proliferation, platelet aggregation, release of coagulation factors, and glycogenolysis. A large volume of literature has demonstrated that AVPR1A contributes to a range of social behaviors in lower vertebrates and humans [37]. But evidence on the association between AVPR1A and cancer is rare. Nor did we find evidence of the link between AVPR1A and thyroid cancer.

Using data mining based on bioinformatics tools, the present study has confirmed CHRDL1, GNAI1, GNA14 and AVPR1A are associated with thyroid cancer although their specific biological functions have not been explored by the molecular biology methods.

For the results of the pathway analysis in this study, we consulted the literature and retrieved more relevant information. ECM-receptor interactions have been found to play a role in the development of papillary thyroid carcinoma [38]. Activation of the p53 signaling pathway could induce apoptosis and lead to tumorigenesis [39]. Zhao P et al. found that retinol metabolism might play a key role in thyroid-associated ophthalmopathy (TAO) (Zhao et al. 2015). West J. et al. found that anomalies in the TGF-*β* signaling pathway seemed to participate in the oncogenesis of thyroid follicular carcinoma (West et al. 2000).

In addition, we used the CMap database to find small molecules of drugs that might reverse the expression of thyroid cancer genes. This discovery will help develop new targeted drugs for thyroid cancer. Adrenosterone (enrichment value -0.914) is an endogenous steroid hormone used as a dietary supplement to reduce body fat and increase muscle mass. It is proposed that adrenosterone, which is primarily responsible for reactivation of cortisol from cortisone, may be an inhibitor of the 11beta-hydroxysteroid dehydrogenase type 1 enzyme (11beta-HSD1) [40]. Hexamethonium bromide (enrichment value -0.813), a non-depolarizing ganglion blocker and an nAChR antagonist, can be used to treat hypertension and duodenal ulcers [41, 42]. After reviewing the literature, we found that the effectiveness and safety of adrenosterone and hexamethonium bromide in thyroid cancer treatment have not been studied. Therefore, further research is urgently needed to reveal the potential of these small molecules in treating thyroid cancer.

In summary, the 7 key genes obtained from the PPI network are closely related to tumorigenesis and tumor progression, suggesting that these genes may be prognostic markers and therapeutic targets of thyroid cancer. At the same time, the KEGG pathway analysis of DEGs provides a new perspective for elucidating the pathogenesis and diagnosis of thyroid cancer, and a new direction for developing targeted inhibitors for thyroid cancer. The present study is based on big data, therefore, these thyroid cancer-associated signaling pathways and key genes need further validation in clinical samples using methods of molecular biology such as RT-PCR and Western blot.

## Figures and Tables

**Table 1:** Details of the thyroid cancer data in GEO.

Reference	Sample	GEO	Platform	Normal	Tumor	Number of DEGs
He H et al. [6]	Thyroid	GSE3467	GPL570	9	9	455
Missing	Thyroid	GSE3678	GPL570	7	7	487
Tomás G et al. (2012), Dom G et al. [5]	Thyroid	GSE33630	GPL570	45	60	904
Pita JM et al. [4]	Thyroid	GSE53157	GPL570	3	24	93

Abbreviation:GEO, gene expression omnibus.

**Table 2:** Integrated DEGs in THCA.

DEGs	Gene names
Upregulated	PRR15 ZCCHC12 LRP4 CITED1 ARHGAP36 LRRK2 KCNJ2 DPP4 QPCT CLDN1 METTL7B FN1 PCSK2 LPAR5 CDH3 SCEL GABRB2 SYTL5 IGSF1 ENTPD1 TENM1 LIPH GALNT7 SERPINA1 LAMB3 FRMD3 C4orf48 MPZL2 PSD3 PROS1 NELL2 RXRG LAMP3 RYR1 GOLT1A SYTL1 SCG5 CHI3L1 NMU HEY2 GABBR2 CAMK2N1 COMP THRSP TIAM1 SLC34A2 AGR2 FAM20A ITGA2 FAXC CFI KLHDC8A TMPRSS4 CYP1B1 ZMAT3 TNFRSF12A UPP1 PDZK1IP1 AMIGO2 SLIT1 NFE2L3 NRCAM GGCT RAB27A EPS8 AX748273 SPOCK1 CCND1 MET CDH2 DCSTAMP SPP1 DTX4 BID NGEF APOC1 NR2F1-AS1 KRT80 RASD2 UHRF1 CORO2A UBE2QL1 DEPDC1B CKS2 ALOX5 PLAG1 C19orf33 SFTPB DUSP4 CD55 DUSP6 NPC2 CLDN10 TBC1D2 NRIP1 DUSP5 TMEM163 LOC100507165 TNC MLLT11 ADAMTS9 SDC4 SHROOM4 TUSC3 ETV5 MUC1 TNIK CHST2 COL13A1 STK32A PDLIM4 APOE KLK10 ALDH1A3 C2CD4A CPNE4 PMAIP1 GALE KCNN4 DIRAS3 PNP TIMP1 LPL TGFA RP6-99 M1.2 NAB2 CTSS CBLN1 SLC27A6 PRSS23 CDKN1A CDKN2B SLPI CTSC SLC35F2 KISS1R LAMP5 CRLF1 NOX4 PLAU KRT19 IER5L KLK7 PDZRN4 SDK1 MRC2 BEAN1 FLRT3 DDB2 RP11-476D10.1 LEMD1 MIR34A LONRF2 GDF15

Downregulated	TFF3 PKHD1L1 MPPED2 LRP1B CDH16 IPCEF1 TCEAL2 TPO PLA2R1 CCL21 DIO1 GPM6A CFD SLC26A4-AS1 SERTM1 DPP6 COL9A3 GHR PROM1 GJB6 DPT OGDHL PAPSS2 LOC646736 ZFPM2 TDRD9 SMOC2 CWH43 SLC26A4 ADH1B KIT IGFBPL1 TNFRSF11B CSGALNACT1 RASSF9 IP6K3 DLG2 HGD SPX WDR72 COL23A1 HSD17B6 FAM167A GDF10 SCN3A TFCP2L1 CRABP1 GPR83 TMEM171 FAM3B EDN3 OGN MUM1L1 EFEMP1 OTOS C11orf74 ZMAT4 ELMO1 AADACP1 GLT8D2 SGK223 LOC440934 MAMDC2 FHDC1 RASSF6 ROR2 MMRN1 FABP4 LMOD1 SDPR FHL1 PTCSC1 TBC1D4 MT1M SLC26A7 MT1F PKIA IMPA2 LOC101929480 TMEM178A LYVE1 PBX4 RELN AVPR1A AIF1L LIFR FGF13 DIRAS2 TCEAL7 LTF FMOD TGFBR3 SLC1A1 FGL2 RNF150 DGKI AKR1C3 ANGPTL1 DEPTOR AOX1 CHCHD10 PLSCR4 AF070581 FXYD6 CITED2 PPARGC1A DYNLRB2 FOLR1 AGR3 KIAA1324 TMEM139 RYR2 CCDC146 TCF7L1 FCGBP HBB RNF157-AS1 GRAMD2 MT1H PLA2G7 APOD CLCNKB GNA14 HSD11B2 SMAD9 SLITRK5 DIO2 MYOC LINGO2 FBLN5 AKAP12 PRDM16 ST7-AS1 PAK3 RAP1GAP FBLN7 RP6-24A23.7 BEX1 SORBS2 TSPAN7MFAP4 LRP2 PCP4 TFPI PRTG CPQ CHRDL1 EYA2 SLC16A2 ANKRD18A CNTN3 FREM2 IYD DNALI1 CCL28 SLC25A15 KCNIP4 BMP8A PTX3 SLC4A4 BTBD11 CRYAB CUX2 MDH1B SELENBP1 EPHA3 SYNM RP13-20 L14.1 RGS16 ITM2A STEAP2 GNAI1 PEG3 PRR15L IRS1 DCN

**Table:** (a) Upregulated genes top 9 enriched GO terms

Category	Term	Count	*P*Value	Adj.*P*-value
Upregulated				
GOTERM_CC_DIRECT	GO:0005576~extracellular region	33	6.02E-07	6.53E-05
GOTERM_CC_DIRECT	GO:0005615~extracellular space	30	4.51E-07	9.78E-05
GOTERM_CC_DIRECT	GO:0070062~extracellular exosome	43	1.38E-05	9.94E-04
GOTERM_CC_DIRECT	GO:0016324~apical plasma membrane	11	9.61E-05	0.005198329
GOTERM_CC_DIRECT	GO:0033116~endoplasmic reticulum-Golgi intermediate compartment membrane	6	1.41E-04	0.006102825
GOTERM_CC_DIRECT	GO:0005578~proteinaceous extracellular matrix	9	0.001212652	0.032377398
GOTERM_CC_DIRECT	GO:0030054~cell junction	12	9.49E-04	0.033762537
GOTERM_CC_DIRECT	GO:0009986~cell surface	13	0.001109523	0.033828883
GOTERM_MF_DIRECT	GO:0008201~heparin binding	8	2.89E-04	0.041460423

**Table:** (b) Downregulated genes top 7 enriched GO terms

Category	Term	Count	*P*Value	Adj.*P*-value
Downregulated				
GOTERM_BP_DIRECT	GO:0006590~thyroid hormone generation	5	2.64E-06	0.002657461
GOTERM_BP_DIRECT	GO:0030509~BMP signaling pathway	7	5.37E-05	0.026654296
GOTERM_CC_DIRECT	GO:0005578~proteinaceous extracellular matrix	15	7.80E-08	1.45E-05
GOTERM_CC_DIRECT	GO:0070062~extracellular exosome	50	3.91E-07	2.42E-05
GOTERM_CC_DIRECT	GO:0005615~extracellular space	32	3.46E-07	3.22E-05
GOTERM_CC_DIRECT	GO:0005576~extracellular region	30	9.41E-05	0.004366683
GOTERM_CC_DIRECT	GO:0043235~receptor complex	7	7.88E-04	0.028910743

(a) Top 9 enriched GO terms of upregulated DEGs. (b) Top 7 enriched GO terms of downregulated DEGs.

**Table:** (a) Pathways of upregulated DEGs

ID	Pathway	Count	*P*-value	Adj.*P*-Val
Upregulated				
KEGG:05222	Small cell lung cancer	9.00	8.62532E-08	6.46899E-06
R-HSA:8952289	FAM20C phosphorylates FAM20C substrates	9.00	3.15636E-07	2.3357E-05
R-HSA:8957275	Post-translational protein phosphorylation	9.00	3.15636E-07	2.3357E-05
R-HSA:1474244	Extracellular matrix organization	13.00	1.53637E-06	0.000110619
WP:2814	Mammary gland development pathway - puberty (stage 2 of 4)	4.00	3.29235E-06	0.000233757
KEGG:04512	ECM-receptor interaction	7.00	5.8434E-06	0.000409038
WP:366	TGF-beta signaling pathway	8.00	1.67948E-05	0.001158841
R-HSA:1474228	Degradation of the extracellular matrix	8.00	2.43709E-05	0.001657223
KEGG:04115	p53 signaling pathway	6.00	3.20579E-05	0.002147877
R-HSA:3000170	Syndecan interactions	4.00	7.37454E-05	0.004867193

**Table:** (b) Pathways of downregulated DEGs

ID	Pathway	Count	*P*-value	Adj.*P*-Val
Downregulated				
R-HSA:209968	Thyroxine biosynthesis	4.00	1.02232E-06	2.2491E-05
R-HSA:209776	Amine-derived hormones	4.00	1.41298E-05	0.000296725
R-HSA:5661231	Metallothioneins bind metals	3.00	9.55155E-05	0.00191031
R-HSA:5660526	Response to metal ions	3.00	0.000206804	0.00392928
KEGG:00350	Tyrosine metabolism	4.00	0.000241586	0.004348555
R-HSA:3560782	Diseases associated with glycosaminoglycan metabolism	4.00	0.000401916	0.006832565
R-HSA:2161282	Elastic fibres bind associated proteins	3.00	0.000452188	0.007235015
R-HSA:1630316	Glycosaminoglycan metabolism	6.00	0.000673119	0.01009678
KEGG:04978	Mineral absorption	4.00	0.000928833	0.013003667
WP:2895	Differentiation of white and brown adipocyte	3.00	0.001220241	0.01586313

(a) Pathways of upregulated DEGs. (b) Pathways of downregulated DEGs.

**Table 5:** Four most significant small molecule drugs.

Rank	CMap name	Mean	Enrichment	*P*	CID
1	Ikarugamycin	0.658	0.955	0.0001	54680304
2	Adrenosterone	-0.679	-0.914	0.0001	223997
3	Hexamethonium bromide	-0.627	-0.813	0.00056	5938
4	Clofazimine	0.525	0.805	0.00066	2794

## Data Availability

The data used to support the findings of this study are included within the article.

## Supplementary Materials

Supplementary Materials The clinical information of data sets.
